# Outcome of vulvar reconstruction in patients with advanced and recurrent vulvar malignancies

**DOI:** 10.1186/s12885-015-1792-x

**Published:** 2015-11-05

**Authors:** Wei Zhang, Ang Zeng, Jiaxin Yang, Dongyan Cao, Xiaodong He, Xiaojun Wang, Yan You, Jie Chen, Jinghe Lang, Keng Shen

**Affiliations:** 1 Departments of Obstetrics and Gynecology, Peking Union Medical College (PUMC) Hospital, Chinese Academy of Medical Sciences & Peking Union Medical College, Shuaifuyuan No.1,Dongcheng District, Beijing, 100730 China; 2 Departments of Plastic Surgery, Peking Union Medical College (PUMC) Hospital, Chinese Academy of Medical Sciences & Peking Union Medical College, Beijing, China; 3 Departments of General Surgery, Peking Union Medical College (PUMC) Hospital, Chinese Academy of Medical Sciences & Peking Union Medical College, Beijing, China; 4 Departments of Pathology, |Peking Union Medical College (PUMC) Hospital, Chinese Academy of Medical Sciences & Peking Union Medical College, Beijing, China

**Keywords:** Vulvar cancer, Vulvar reconstruction, Quality of life, Complications

## Abstract

**Background:**

The use of flaps in vulvar cancer-related reconstruction has been increasing, but few studies have evaluated the outcome and quality of life of patients after this surgery. The purpose of this study was to evaluate the outcomes of vulvar reconstruction using musculocutaneous/skin flaps in patients with advanced and recurrent vulvar malignancies.

**Methods:**

Patients with vulvar malignancies who underwent vulvar reconstruction using different types of flaps were retrospectively reviewed. Patient outcomes were evaluated with a focus on quality of life and prognosis.

**Results:**

Thirty-six patients were enrolled, 58.33 % of them used anterolateral thigh flap (ALT), 16.67 % of them used pudendal thigh flap (PTF), 11.11 % of them used deep omferior epigastric perforator (DIEP) and gracilis myocutaneous flap were used in 2.78 % of the patients, the other 11.11 % patients used two types of flaps. Eleven patients (30.56 %) developed complications, including 5 patients (13.89 %) with partial necrosis, 5 (13.89 %) with minimal wound dehiscence and 1 (2.78 %) with flap cellulitis. All patients who developed partial necrosis (13.89 %) underwent reoperation. The mean verbal rating scale score was 1.44 before reconstruction and 0.17 after surgery (*P* < 0.0001). The mean performance status was 1.67 before surgery and improved to 0.31 after surgery (*P* < 0.0001). The median overall follow-up time after vulvar reconstruction was 9 months. Twenty-one patients (58.3 %) developed recurrence at a median interval of 5 months after vulvar reconstruction. After a median follow-up time of 14 months, 41.7 % of the patients were living and disease-free. The 5-year survival of the 36 patients was 53.8 %.

**Conclusion:**

Soft tissue reconstruction in patients undergoing resection of advanced/recurrent vulvar malignances is associated with a low rate of postoperative complications, decreased pain, and improved functional status. Although the recurrence rate in this patient population is high, a reasonable proportion of patients who undergo resection for advanced/recurrent vulvar cancer and reconstructive surgery appear to benefit.

## Background

Surgery is the mainstay treatment for vulvar malignancies. However, vulvar malignancies often have a high risk of relapse that may reach 65 % at the scheduled follow-up [1]. Local recurrence is more common than distant metastasis in patients with larger tumors [2], and can be successfully treated by tumor excision or irradiation. However, multiple surgeries and radical excision often leaves a large defect without sufficient tissue for coverage, which delays wound healing and increases post-operative morbidity. All of these factors have an adverse impact on the patient’s quality of life (QoL), which is generally accepted as an important outcome parameter, in addition to the long-term survival, mortality and complication-related morbidity [3]. Therefore, vulvar reconstruction should be considered after radical surgical treatment to reduce the morbidity and improve the patient’s QoL.

In recent years, the use of myocutaneous/muscle flaps for reconstruction has increased in the treatment of gynecologic malignancies [4–6]. However,few studies have evaluated the outcomes and QoL of patients who have undergone this surgical treatment. In the present study, we evaluated the outcomes of different types of flaps used for vulvar reconstruction in patients with advanced and recurrent vulvar malignancies, focusing on the complications related to the flaps and on the QoL and survival of patients.

## Methods

From 1998 to 2013, patients with advanced and recurrent vulvar malignancies underwent vulvar reconstruction using myocutaneous or skin flaps at the Department of Obstetrics and Gynecology, Peking Union Medical College Hospital. The patients’ outcomes were evaluated. Clinical data were collected and reviewed by searching the medical records, operative notes, hospital discharge records and outpatient clinic follow-up records. The histopathological diagnosis was made and then reviewed by two experienced pathologists. All 36 patients were re-staged according to the revised International Federation of Gynecology and Obstetrics (FIGO) stage 2009. Advanced vulvar cancer was defined as a FIGO stage of ≥ III in this study. The study was approved by the ethics committee at Peking Union Medical College Hospital, Beijing, China. Informed consent was obtained in written from the all the participants in our study.

### Surgical technique

The surgery plan was established by preoperative and intraoperative consultation with plastic surgeons. Gynecologic oncologists, urologists and colorectal surgeons performed extirpative surgery before reconstruction. The surgical margin was confirmed to be negative by frozen section during the surgery before reconstruction. Once the final defect was known, the plastic surgeon began to evaluate the size and extent of the defect to design the flaps for reconstruction. The laxity and quality of the perineal skin were also evaluated. After careful evaluation, different flap types were used for reconstruction. An anterolateral thigh (ALT) flap was designed and harvested as previously described using a protocol suggested by our team [4]. The other four flap types including pudendal thigh flap (PTF), deep inferior epigastric perforator (DIEP) flap, gracilis myocutaneous flap and transverse rectus abdominis musculocutaneous (TRAM) flap, were also harvested and used as previously described [7–9]. The selection of different types of flaps was based on the patients’ history, including prior irradiation, history of vulvar surgery, history of cesarean section, and nature of vulvar defects.

### Post-operative care

All patients were required to undergo bed rest for 5 to 7 days after the operation and maintain hip-flexion and the genuflecting position to relieve the pressure on the flaps. The patients were then encouraged to participate in bedside walking. The plastic surgeon carefully checked the color and temperature of the flap every day until flap survival was confirmed. The Foley catheter remained in place for at least 7 days. Patients with close surgical margins [10] , high-risk factors [10, 11] and patients with negative surgical margins according to frozen section during surgeries that turned into positive in post-operative pathological examinations were given post-operative adjuvant treatment.

### Follow-up plan

After discharge, a gynecologic oncologist and plastic surgeon followed up the patients at an outpatient clinic 1-month after surgery. The patients were then followed at the gynecologic oncology clinic every 3 months for the first 2 years and then every 6 months for 3–5 years. The follow-up evaluation involved a complete history, physical and gynecological examination, laboratory examination, and pelvic and abdominal ultrasonography. Biopsies were performed if recurrence was suspected. Local recurrence was confirmed by pathological examination, and distant metastasis was diagnosed by positron emission tomography-computed tomography (PET-CT) and/or computed tomography (CT). Progression-free survival (PFS) was defined as the time interval from the date of vulva reconstruction to the date of disease progression or recurrence. Overall survival (OS) was defined as the time interval from the date of the primary surgery to the date of death or last contact. The follow-up deadline was 30 June, 2014.

### QoL and post-operative complications

The QoL assessment focused on disease-specific pain and performance status. The degree of pain was evaluated with a four-category verbal rating scale (VRS-4)( 0 = no pain, 1 = mild pain, 2 = moderate pain, and 3 = severe or intense pain) [12, 13]. The performance status was determined using the Eastern Cooperative Oncology Group/World Health Organization/Zubrod (ZUBROD-ECOG-WHO) scale (0 = normal activity; 1 = symptoms, but nearly ambulatory; 2 = some bed time, but needs to be in bed for < 50 % of the normal daytime; 3 = needs to be in bed for > 50 % of the normal daytime and 4 = unable to get out of bed) [14]. The VRS-4 and performance status were evaluated before surgery and 1 month after surgery.

Plastic surgeons evaluated flap-associated complications during the hospital stay and after discharge from the hospital at the outpatient plastic surgery department. Postoperative complications were defined as major or minor according to a previous study [15]. Major complications included total or partial flap necrosis, major wound of dehiscence more than one- third of the incision length,and persistent dead space. Persistent dead space is defined as dead space requiring a supplementary reconstructive procedure during the follow-up period. Minor complications included minor dehiscence of less than one-third of the incision length that healed after debridement [15]. Hematoma, seroma, cellulitis and abscess were also considered complications. In this study, we considered complications requiring reoperation as major complications and those requiring debridement or dressing changes as minor complications. Necrosis was defined as clinical evidence of dead tissue due to circulatory ischemic factors [6]. Dehiscence was defined as the separation of surgical margins [6].

### Statistical analysis

The patient age, operation time, length of hospitalization, flap sizes, time of follow-up, survival curve and 5-year survival were statistically analyzed by SPSS software version 13.0 (SPSS, Inc., Chicago, IL, USA). Kaplan–Meier survival plots and Student’s two-tailed t-test were used for paired data; the independent samples t test was also used. A P value of <0.05 was considered statistically significant.

## Results

### Patients’ characteristics

**Fig. 1:**
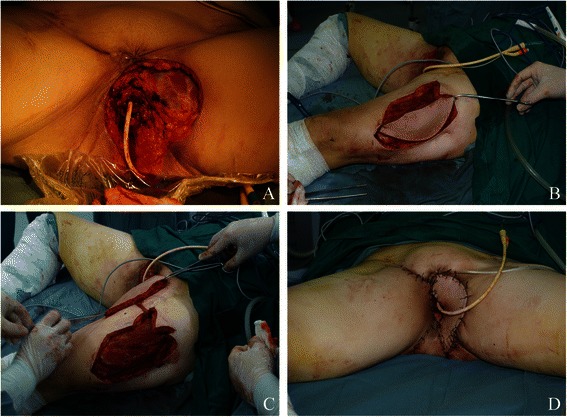
The patient underwent vulvar reconstruction for recurrent squamous cell carcinoma (SCC) after tumor resection. **a** Flap design, **b** Perforator dissection (**c**) Detection of the perforators and flap design (D) Appearance of the vulvae after reconstruction

Forty flaps were performed in 36 patients. Basic demographic and clinical information are shown in Table 1, including age, FIGO stage, histological type, time of reconstruction, prior irradiation, and flap type. The mean age of the patients was 49 years. The main histological tumor type was squamous cell carcinoma (72.22 %). Seven patients underwent vulvar reconstruction at the time of primary treatment for advanced vulvar malignancies. Twenty-nine patients underwent vulvar reconstruction after surgery for tumor recurrence (Fig. 1). Twenty of the 36 patients (55.56 %) received chemo-irradiation or radiation before vulvar reconstruction.

**Table 1:** Basic demographic and clinical information of the 36 patients

Characteristics	Number (%)
Mean age(year)	49.7 ± 13 (23–74)
Mean BMI	24.17 ± 4.64 (18.66–36.13)
FIGO stage	
I	7 (19.44 %)
II	5 (13.89 %)
ΙΙΙ	12 (33.33 %)
ΙV	2 (5.56 %)
Unstaged	10 (27.78 %)
Histology	
Squamous cell carcinoma	26 (72.22 %)
Melanoma	3 (8.33 %)
Bartholin gland carcinoma	2 (5.56 %)
Sarcoma	2 (5.56 %)
Others	3 (8.33 %)
Time of reconstruction	
Primary treatment	7 (19.44 %)
After recurrence	29 (80.56 %)
Previous radiation	
Yes	20 (55.56 %)
No	16 (44.44 %)
Types of skin flap	
Anterolateral thigh flap(ALT)	21 (58.33 %)
Pudendal thigh flap(PTF)	6 (16.67 %)^a^
Deep omferior epigastric perforator (DIEP)	4 (11.11 %)
Gracilis myocutaneous flap	1 (2.78 %)
Two types of skin flaps	4 (11.11 %)
Characteristics of defects	
Unilateral	6
Bilateral	30
Composite defects	
Involving the vaginal canal	7
Involving the urethral canal	4

^a^SCC squamous cell carcinoma, *PENT* primitive neuroectodermal tumor, *MEHE* malignant epithelioid hemangioendotheliom

Thirty-two patients had only one skin flap type and four patients had two different types. Among patients with a single skin flap type, the ALT flap was the most commonly used flap (21/36 cases, 58.33 %). Four patients had two flap types: an ALT flap combined with a DIEP flap, TRAM flap or gracilis myocutaneous flap; and one had a DIEP flap combined with a PTF.

The mean operation time among all 36 patients was 338 min (range, 120–660 min) with a mean estimated blood loss of 342 ml (range, 50–2000 ml). The flap size, operation time and blood loss associated with each different flap are shown in Table 2. The median length of hospitalization was 31 days (range, 13–145 days).

**Table 2:** Comparison of different flap parameters

Flap parameters	ALT	DIEP	PTF	Gracilis Flap	
Flap size (cm^2^)	179.21 ± 108.73	151 ± 67.12	70 ± 24.72	84	
Operation time (min)	371.25 ± 38.72	300 ± 129.6	280.2 ± 140.4	240	
Blood loss (ml)	278.13 ± 317.79		575 ± 403.1	175 ± 781.9	100

*ALT* anterolateral thigh, *PTF* pudendal thigh flap, *DIEP* deep inferior epigastric perforator

### Improvement in QoL and post-operative complications

All 36 patients had one or more symptoms that significantly influenced their QoL before reconstruction, including 23 (63.90 %) with pain, 3 (8.00 %) with pruritus, 18 (50.00 %) with ulceration, 6 (16.67 %) with edema, and 6 (16.67 %) with vulvar hemorrhage. Five patients (13.89 %) could not sit, sleep or walk without analgesics, and one patient was unable to get out of bed. The tumor-related symptoms in the other 30 patients (83.33 %) were completely removed. After surgery, no patient felt pruritus, ulceration,edema or hemorrhage. For 23 patients suffered pain before surgery, 17 patients were completely relived and 6 patients (16.67 %) still felt slight pain. The mean VRS-4 score among all 36 patients was 1.44 before reconstruction and decreased to 0.17 after surgery (*P* < 0.0001). The mean performance status was 1.67 before surgery and improved to 0.31 after surgery (*P* < 0.0001) (Table 3).

**Table 3:** Mean VRS-4 and mean performance status before and after surgery

	Before surgery	After surgery	*P* value
Mean VRS-4	1.44 ± 0.88	0.17 ± 0.34	*P* < 0.0001
Mean Performance status	1.67 ± 0.89	0.31 ± 0.89	*P* < 0.0001

*VRS-4* four-category verbal rating scale

None of the patients experienced complete flap loss. Eleven patients (30.56 %) developed complications, including 5 patients (13.89 %) with major complication and 6 patients (16.67 %) with minor complication. All patients who developed partial necrosis (13.89 %) underwent reoperation. More specifically, 5 patients (13.89 %) suffered partial necrosis, 5 (13.89 %) with minimal wound dehiscence and 1 (2.78 %) with flap cellulitis. All patients who developed partial necrosis (13.89 %) underwent reoperation. The most common symptoms of partial necrosis were a pale flap color and low flap temperature of flap followed by cyanosis, blisters, or swelling. Most cases of minimal dehiscence were found after removing the sutures. Among the patients with major complications, four had an ALT flap and one had a PTF flap. Among the patients with minor complications, three had an ALT flap and one each had a TRAM flap, DIEP flap and PTF. All patients who developed partial necrosis underwent reoperation, and five patients with minimal wound dehiscence were treated with dressing changes. The case of cellulitis resolved after debridement and dressing changes. All wounds healed favorably after appropriate treatment. Twenty-one patients received adjuvant therapies, such as chemotherapy and radiotherapy. Among patients who received chemotherapy, the mean time interval between surgery and adjuvant therapy was 16 days. Among patients who received radiotherapy, the mean interval was 29 days. The reconstructed vulvae were plump and elastic. The donor sites healed without functional impairment, and no donor site morbidity was reported. No late complications were recorded. One patient with a PTF reported perineal strain upon sitting.

### Follow-up results

**Fig. 2:**
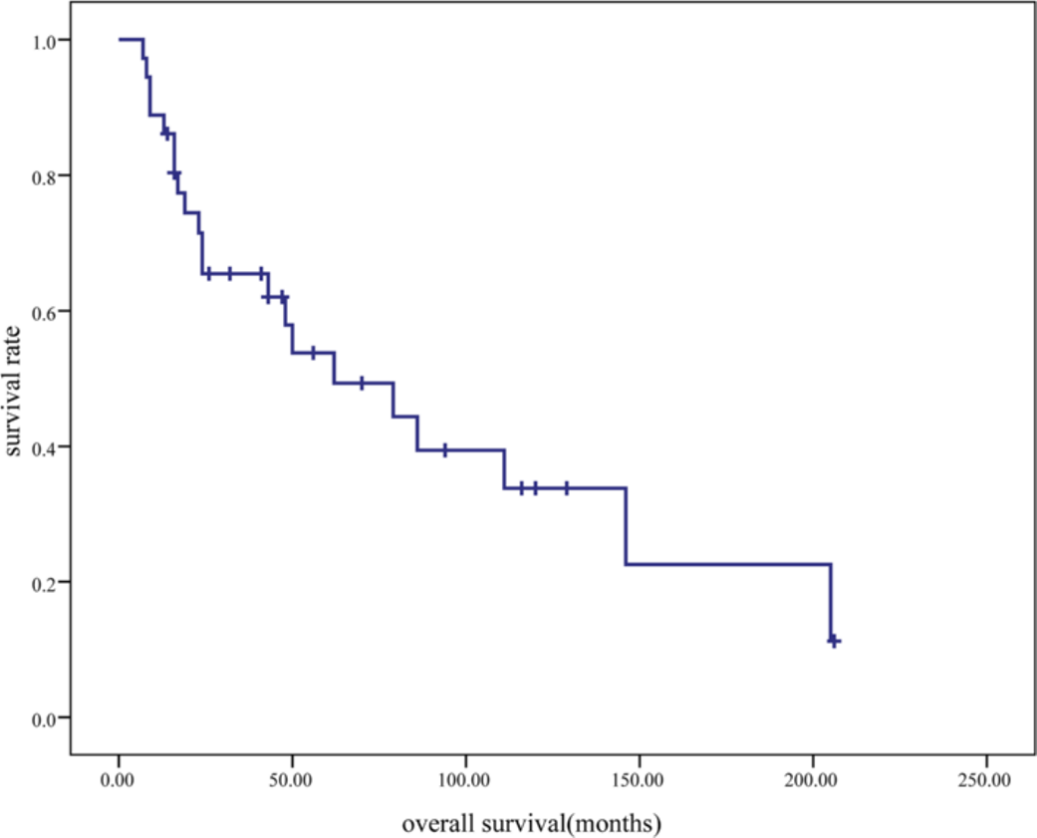
Kaplan-Meier survival curve showing the survival of the 36 patients

The median follow-up duration was 9 months (range, 1–75 months) after vulvar reconstruction. Twenty-one patients (58.33 %) were confirmed to have relapsed after vulvar reconstruction. Sixteen patients had local recurrence, five developed distant metastasis to the bilateral lungs or liver. The other 15 patients were living and disease-free at a median follow-up of 14 months (range, 1–60 months). Of all 36 patients, 21 patients (58.33 %) had died at the last time of contact; 20 of them died of recurrence and 1 died of myocardial infarction. The median overall follow-up time was 43 months (range, 7–206 months). The median progression-free survival (PFS) was 5 months, and the median overall survival was 62 months. The 5-year survival rate of the 36 patients was 53.8 % (Fig. 2).

## Discussion

Commonly used reconstructive options include skin grafts, local skin flaps and myocutaneous flaps [16]. Each of these has advantages and limitations in different reconstruction situations. For example, skin grafts are most useful for superficial defects, but their use is limited in patients with major resections. Additionally, local flaps and regional flaps have been described in many vulvar reconstructions, including V-Y flaps [6], inferior gluteal flaps [17], and lotus petal flap [18, 19]. Application of these flaps in vulvar reconstruction has produced good outcomes with short hospital stays and low complication rates. However, most such flaps are relatively small in size, and their use is influenced by the surgical history of the donor site, especially in irradiated tissue [20, 21]. Myocutaneous flaps include the TRAM flap and gracilis myocutaneous flap. The DIEP flap [22] and ALT flap are perforator skin flaps, while the PTF is a fasciocutaneous flap. However, prior irradiation, a history of surgery, and certain situations may also limit or preclude the use of these flaps. The ALT flap was first described by Song et al. [23] in 1984. It is a large skin flap based on the septocutaneous artery flap concept that can safely be harvested and provide sufficient tissue to fill large defects [4, 24, 25]. Because of its reliability and versatility, it has been successfully used in many soft tissue reconstructions, such as those in the head and neck region [26, 27]; however, there are relatively few descriptions of the use of ALT flaps for treatment of gynecologic malignancies.

For advanced and locally recurrent vulvar malignancies, surgical treatment, which provides acceptable survival results, is still the most common treatment [20–22]. Although radical surgery can completely remove the tumor, it is associated with complications such as wound infection, delayed wound healing, and an increased hospital stay. Successful wound healing is critical to minimizing patient morbidity and ensuring that adjuvant therapies can be performed without delay. Additionally, vulvar surgery often introduces problems with regard to QoL and body image.[23]. Therefore, oncological vulvar reconstruction is necessary to improve surgical outcomes and QoL.

Many studies have focused on different methods of vulvar reconstruction, such as those using skin grafts, local skin flaps or regional skin flaps [6, 24, 25]. The most commonly used flaps are V-Y flaps [6], lotus petal flaps [18] and PTF. The choice of surgical approach and flap type depend on many factors, such as the patients’ prior treatment and surgery, which need to be carefully reviewed when formulating a reconstructive plan. Patients who have received radiotherapy often exhibit poor vascularization in the radiation field which limits the use of local and regional flaps.

In this study, we evaluated a consecutive series of 36 patients treated with 5 types of flaps for oncological vulvar reconstruction. The majority of patients had recurrence or a history of radiotherapy. Because of their treatment history and disease status, there was little use of local flaps; all patients underwent repair with regional skin flaps. Staiano et al. [25] published a series on vulvar reconstruction; most of the patients in their study had received radiotherapy. There were minor complications in 40 % of patients, and a local flap was used in 10 patients [25]. By comparison, although most of the patients in our study received radiotherapy, there were minor complications in only 13.89 % of the patients. This may be because all of the patients had a regional flap that was less influenced by prior irradiation. Additionally, no functional impairment occurred in our study. Hence, for patients with a history of irradiation, flap reconstruction is a safer option, especially that using a distant pedicle flaps. This method may offer a better chance of healing.

Patients with vulvar cancer are often in pain, which influences the patients’ QoL. In this study, all patients’ VRS-4 score and performance status were evaluated before and after surgery. The mean VRS-4 score was 1.44 before reconstruction and 0.17 after surgery (*P* < 0.0001). The performance status was 1.67 before surgery and improved to 0.31 after surgery (*P* < 0.0001). Most of the tumor-related symptoms were gone after surgery. Our results demonstrate that vulvar reconstruction significantly improved the patients’ QoL, which is very important for patients with advanced and recurrent diseases.

In previous studies, the survival rates of patients with vulvar carcinoma and positive inguinal lymph nodes ranged from 21 to 53 % [26–28]. Although most patients were in the late stage and had recurrent tumors, the 5-year survival rate of our 36 patients reached 53.8 %. This demonstrates that with good pre-operative evaluation and post-operative management, patients with advanced and local recurrence can benefit from radical excision and vulvar reconstruction.

Our experience with the present a series of cases allowed us to develop a reconstructive approach for different vulvar defects. The choice of surgical approach and flap type depends on many factors. A careful review of patients’ conditions and prior histories is needed. In the present study, defects were defined as simple defect (localized soft-tissue deficiencies) and complicated defects (irradiated defects or extensive soft-tissue deficiencies). Simple defects can be reconstructed with local flaps or ALT flap. For bilateral vulvar defects, fenestrated or split ALT flaps also work well. For complicated defects, ALT flaps can be elevated with customized dimensions and components tailored to each defect [4]. For mons pubis defects, the DIEP flap and groin flap are better choices.

Our study has some limitations, such as the lack of information about sexual function because most patients are unwilling to share this information. Sexual function after vulvar reconstruction is a very important factor in assessing improvement in QoL and is worthy of further investigation.

## Conclusion

The use of myocutaneous/skin flap for vulvar reconstruction after removal of advanced or recurrent vulvar malignances is associated with a low rate of postoperative complications decreased pain and an improved functional status. Although the recurrence rate in this patient population is high, a reasonable proportion of patients who undergo resection for advanced/recurrent vulvar cancer and reconstructive surgery appear to benefit.

## Abbreviations

ALT: Anterolateral thigh
DIEP: Deep inferior epigastric perforator
PTF: Pudendal thigh flap
TRAM: Transverse rectus abdominis musculocutaneous
VRS: Verbal rating scale
QoL: Quality of life
FIGO: International Federation of Gynecology and Obstetrics
PET-CT: Positron emission tomography-computed tomography
CT: Computed tomography
PFS: Progression-free survival
OS: Overall survival
SCC: Squamous cell carcinoma
PENT: Primitive neuroectodermal tumor
MEHE: Malignant epithelioid hemangioendotheliom
